# Notes and News

**Published:** 1893-07-15

**Authors:** 


### Notes and News.

Collected and Communicated.

Baron Henry de Worms presided last week at the annual distribution of prizes to the students of the Charing Cross Hospital Medical School. The Dean referred to the Royal Commission on the subject of a Teaching University for London, and said it was likely to result in the development of a great university such as London ought to possess.

H.R.H. The Duke of York has received an interesting present from the staff and sick sailors in the Dreadnought Hospital, Greenwich (the chief hospital of the Seamen's Hospital Society). The present consists of a ship's barometer, mounted in silver, and engraved with a model of the old "Dreadnought," and it bears the following inscription: "From the Staff and Seamen of all Nations in the Dreadnought Hospital to H.R.H. the Duke of York, K.G., 'The Sailor Prince,' on his Marriage, 6th July, 1893." H.R.H. is vice-patron of the Seamen's Hospital Society, and as a sailor has taken an active interest in this national marine charity. The barometer has been manufactured by Messrs. Mappin and Webb, of Queen Victoria Street, E.C.

The Smithsonian Institution of America in compliance with the conditions attending a donation from Mr. Thomas Hodgkins, of New York, now issues a list of prizes for treatises relating to the atmospheric air in connection with the welfare of man. Four prizes are offered: One of 10,000 dols., two of 2,000 dols., three of 1,000 dols., and the last will consist of a gold medal. The lines on which the treatises are to be written are definitely set forth, and they may be English, French, German, or Italian, and all save the first on the list must be delivered before July 1st, 1894. It is probable that special grants of money may be made to specialists engaged on original investigations upon atmospheric air and its properties. All communications should be addressed to S. P. Langley, Secretary, Smithsonian Institute, Washington, U.S.A.

Last week the Prince and Princess of Wales opened a bazaar in aid of the Alexandra Hospital for Children with Hip Disease, at the Westminster Town Hall. The Princess of Wales and her daughters were presented with bouquets by the little patients.

Lady Meath's interest in many useful branches of charity is well known. These include the Girls' Friendly Society and the care of epileptics. Both these and the Brabazon Home for Invalids, benefited by a *pot pourri* opened at St. Martin's Town Hall by the Princess Christian on the 28th ult. The stall-holders included a large number of distinguished personages. The Bishop of Marlborough gave a warm and eloquent address. We sincerely hope that the various institutions interested received substantial assistance on the occasion.

Not very long ago we had the pleasure of visiting the Meath Home for Epileptics. We cannot do better than advise our readers to follow our example. The Home is beautifully situated in the charming village of Godalming, and is conveniently near to the station. The Home looks what it once was, a delightful home-like country house, standing in its own grounds, part of which are laid out in terrace walks on a gentle slope. On entering we were struck with the fresh and cheery look of everything within. Lady Meath has taken the utmost personal interest in the arrangements, and the furniture was especially made for her by Messrs. Shoolbred. Lady Meath has expended over £4,000 on this excellent scheme, which provides for epileptic women and girls, those belonging to the G.F.S. standing first for admission, and being charged a smaller sum for maintenance, though the payments under any circumstances are exceedingly small, and £500 per annum is required in subscriptions to cover the working expenses.

Sir William Broadbent, Bart., has received the unanimous and warm congratulations of the profession upon his selection by the Queen for the honour of a baronetcy. Sir William Broadbent possesses qualities which are specially needed in a leader of the medical profession at the present time. It is notorious that a widespread feeling of dissatisfaction exists throughout the profession, from the narrowness of view which often marks the deliberations of the Council of more than one of the great corporate bodies. Fortunately Sir William Broadbent is not only a physician of eminence, but he is a man with a backbone. If he believes a thing to be right his whole career shows that that thing will be done, or Sir William will know the reason why. We welcome the honour which has been conferred upon Sir William Broadbent, because we believe it to be fraught with good to the whole profession. Sir William Broadbent holds most enlightened views on many questions, possesses the courage of his convictions, and the measure of his popularity is surpassed by that of no other member of the great profession he adorns. In offering him our congratulations, we wish Sir William a long and brilliant career of happy, courageous, and productive labour, full of benefit to his colleagues, his family, and the public at large.

